# Whole genome sequencing identifies variants associated with sarcoidosis in a family with a high prevalence of sarcoidosis

**DOI:** 10.1007/s10067-021-05684-w

**Published:** 2021-04-27

**Authors:** Daan Fritz, Bart Ferwerda, Matthijs C. Brouwer, Diederik van de Beek

**Affiliations:** Department of Neurology, Amsterdam Neuroscience, University of Amsterdam, Amsterdam UMC, P.O. Box 22660, Meibergdreef 9, 1100 DD Amsterdam, The Netherlands

**Keywords:** All genetics, All immunology, Association studies in genetics, Case-control studies

## Abstract

**Objective:**

We studied genetic risk factors associated with sarcoidosis within a family with a high prevalence of this disease.

**Methods:**

We studied 41 members of a family with a high rate of sarcoidosis, including an index patient with treatment-resistant neurosarcoidosis. Whole genome sequencing was performed for six affected family members and variations associated with loss of function were filtered out as candidate genes. Findings were validated by using amplicon sequencing within all 41 family members with DNA available and candidate genes were screened on absence and presence within the sarcoidosis affected and non-affected.

**Results:**

Family members (*n* = 61) from 5 generations were available for participation including 13 subjects diagnosed with sarcoidosis (20%). Analyses identified 36 candidate variants within 34 candidate genes. Variations within three of these genes (JAK2, BACH2, and NCF1) previously have been associated with autoimmune diseases.

**Conclusions:**

We identified 34 genes with a possible role in the etiology of sarcoidosis, including JAK2. Our results may suggest evaluation of JAK inhibitors in treatment-resistant sarcoidosis.

**Table:** 

**Key Points** *• JAK2 has a potential role in the etiology of sarcoidosis and is a potential therapeutic target.* *• We identified 33 additional candidate genes of which BACH2 and NCF1 have been previously associated with autoimmune disease.*

**Supplementary Information:**

The online version contains supplementary material available at 10.1007/s10067-021-05684-w.

## Introduction

Sarcoidosis is a granulomatous inflammatory disorder that manifests most commonly between the age of 20 and 40 years [1]. This disease has a prevalence between 5 and 64 per 100,000 with highest incidences in the Northern European and African-American populations [1]. Neurosarcoidosis is a severe variant of sarcoidosis, characterized by granulomas involving the nervous system, with heterogeneous clinical presentation and a high relapse rate [2].

The etiology of sarcoidosis is unknown and it is hypothesized to be caused by an exaggerated immune response to unknown antigens in genetically predisposed individuals [1]. Granuloma formation may be initiated by the innate immune system with a possible role for toll-like receptor 2 [3]. Presentation of antigens through MHC class I or II molecules induces adaptive effector T cell responses, mainly a T helper cell 1 (Th1) and T helper cell 17 response via interleukin 17 (IL-17) and 23 (IL-23) [4]. A genetic basis was shown by a twin study, estimating a heritability of 66% [5]. Genome-wide association studies (GWAS) and candidate gene studies have described candidate genes, including annexin A11 (ANXA11), butyrophilin-like 2 (BTNL2), coiled-coil domain containing 88B (CCDC88B), BCL2-associated agonist of cell death (BAD), and associations in the human leukocyte antigen (HLA), most notably HLA-DRB1 and HLA-DBQ1 [4].

At our outpatient clinic, a tertiary referral center for sarcoidosis, we lost a patient with treatment refractory neurosarcoidosis due to a relapse of the disease. Our patient belonged to a Suriname family with an unusual high rate of sarcoidosis. At the request of our patient and her family, we tried to unravel genetic risk factors associated with sarcoidosis using whole genome sequencing.

## Methods

We constructed a pedigree based on the clinical information provided by the family. Not all information of the fourth and fifth generations could be provided. Diagnosis of sarcoidosis was made by the treating physician, according to international guidelines [1]. Tissue confirmation of non-caseating granulomatous inflammation was preferred but not required for inclusion. We collected data on medication use, diagnosis, organ involvement, treatment response, and outcome. This study was done at the request of the family. All participants provided written informed consent and the study was done according to Dutch legislation.

Saliva of 40 family members was collected for DNA extraction and isolated using the Oragene saliva collection system (OG-500, DNA Genotek) according to manufacturer’s protocol. Additionally, blood was withdrawn from one patient in sodium/EDTA tubes for DNA extraction. Isolation of DNA was performed with the Gentra Puregene isolation kit (QIAGEN) according to the manufacturer’s protocol.

For the whole genome sequencing, DNA concentration was determined by means of fluorometric measurement (Qubit, Thermi) and quality was checked by determining the absence of degradation and presence of high molecular weight DNA. Whole genome sequencing of six sarcoidosis-affected family members was done using the Illumina HiSeq X platform at the Hartwig Medical Foundation DNA sequencing center. Paired-end reads for each individual were merged using Picard (version 1.92) and aligned to the GRch37/HG19 reference genome using the LifeScope aligner (version 2.5.1, Applied Biosystems). To minimize mismatched bases between reads, realignments were performed using the RealignerTargetCreator function in GATK (version 2.7-4). Samples were recalibrated with GATK Recalibrate and variants were called using GATKs HaplotypeCaller (version 3.3-0) with default settings. Variants for analysis were filtered on a minimal read depth of 20 and genotype quality of 99. After filtering, all samples were combined and genotyped by using GATK CombineGVCFs and GenotypeGVCFs (version 3.3-0). Finally, all chromosomal locations for found variants were annotated using SnpEff and the UCSC variant annotation integrator tool (https://genome.ucsc.edu/) [6]. We updated all mentioned locations in the manuscript according to the GRCh38 using Lift Genome Annotations for the UCSC genome browser.

For the validation of candidate variants, AmpliSeq^ؑ^™ (Life Technologies, Carlsbad, CA, USA) custom panels were developed using Ion AmpliSeq™ designer software capturing 307 candidate variants. Libraries were constructed using Ion AmpliSeq™ Library Kit (v2.0, Thermo Fisher) according to the manufacturer’s instructions. Validation and quantification of enriched targeted DNA were performed. Emulsion PCR was performed using the SOLiD EZ Bead Emulsifier and Amplifier (Applied Biosystems). Sequencing was performed on the SOLiD 5500xl sequencer (Life Technologies) generating paired-end reads (50 bp forward and 35 bp reverse).

In the analysis pipeline, we used the six whole genome sequenced affected family members as a template to find variations associated with loss of function of a gene, consisting of variants with frameshift, in-frame indel, start/stop codon change, and missense using Ingenuity Variant Analysis™ (QIAGEN Redwood City, CA). All exomic variants that had a call quality of more than 20 were imported in Ingenuity Variant Analysis™. Filters were set to exclude common variants, remove variants in the top 5% most exonically variable and all variants with an allele frequency of ≥ 1% in the 1000 Genomes, ExAC, and NHLBI ESP databases. We kept variants which were associated with gain of function or which were homozygous, haplo-insufficient, or hemizygous.

Using AmpliSeq™, we verified the Ingenuity Variant Analysis™ results for all six affected family members and 35 other family members for whom we were able to collect DNA. Overall, we performed amplicon sequencing in 41 individuals, including the six whole genome sequenced individuals. Two researchers independently evaluated all AmpliSeq™ results by visually inspecting the reads using Integrative Genomics Viewer (IGV, Broad Institute, Cambridge, MA, USA). Low-quality variants and identical variation between all family members were excluded. Protein function and/or allele frequency difference between affected and non-affected family members was used to further select variations. An independent in-house reference whole exome sequencing database was used to exclude all variants present with a minor allele frequency (MAF) of 0.01. Finally, we manually re-evaluated all population MAF of the remaining variations by consulting ExAC. Furthermore, all variants were evaluated using the combined annotation-dependent depletion (CADD) score and the variants were cross referenced in the ClinVar database.

All remaining genes were thereafter evaluated on function and previously reported roles in sarcoidosis, other granulomatous and/or autoimmune disease, or a role in the immune system. Furthermore, we performed pathway analysis using the online tool from the STRING database. STRING database was used to evaluate if the reported proteins in which our variants were found had any similar interaction networks or functional enrichment analysis.

### Data availability statement

Anonymized data not published in the article is available on request by any qualified investigator.

## Results

**Fig. 1:**
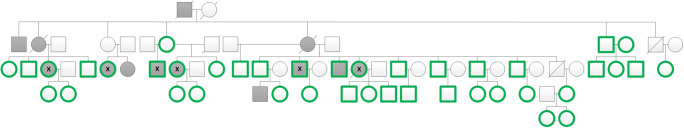
Pedigree of individuals of whom DNA was collected

Family members from 5 generations were available for participation in the study (Fig. 1), consisting of 61 subjects of whom 13 were diagnosed with sarcoidosis (21%). Of these, genetic material could be collected from 41 patients (67%). The family was of African Surinamese ancestry. Characteristics of the 13 affected patients are presented in Table 1. The diagnosis of sarcoidosis was confirmed by pathological examination in 8 of the 13 patients (62%), based on clinical suspicion in combination with imaging in 4 patients and on clinical suspicion only in 1 patient. The latter was a patient with an isolated eye manifestation. Organ system involvement consisted of the lungs in 11 patients, eyes in 3, joints in 3, skin in 2, and nervous system in 2. Treatment was known for 8 patients and consisted of systemic use of immunosuppressive therapy in 5 patients as follows: prednisone in 5, methotrexate in 2, and infliximab, chloroquine, and azathioprine in 1 patient. One patient was treated with prednisolone eye drops only. Treatment response was known for 8 of 13 patients and consisted of complete remission in 7 (88%) and death due to sepsis in the index patient.

**Table 1:** Clinical information of individuals with sarcoidosis

Characteristic	*n*/*N* (%)	Characteristic	*n*/*N* (%)
Age at diagnosis known	6/13 (46)	Treatment	
Age at diagnosis (IQR), years	40 (33–43)	Corticosteroids	5/8 (63)
Mean current age (IQR), years^a^	61 (50–80)	Methotrexate	2/8 (25)
Sex (male)	6/13 (46)	Infliximab	1/8 (13)
Organ system involvement		Azathioprine	1/8 (13)
Lungs	11/13 (85)	Chloroquine	1/8 (13)
Eyes	3/13 (23)	Corticosteroid containing eye drops	1/8 (13)
Joints	3/13 (23)	No treatment	3/8 (38)
Skin	2/13 (12)	Unknown	5/13 (38)
Neurological	2/13 (12)	Outcome	
Diagnosis		Remission	7/8 (88)
Biopsy confirmed	8/13 (62)	Deterioration	1/8 (13)
Suggestive imaging^b^	4/13 (31)	Unknown	5/13 (38)
Clinical diagnosis^c^	1/13 (8)	Death^d^	3/13 (23)

Data are number/number assessed (%) and median (25th–75th percentile)

^a^Age on 01-01-2019

^b^Bilateral hilar lymphadenopathy on chest X-ray in all patients

^c^Uveitis anterior, diagnosis made by an ophtalmologist

^d^Last time of follow-up 01-01-2017. One patient died of deterioration due to disease progression

Whole genome sequencing was done on the 6 patients with sarcoidosis (Fig. 1). These patients had various affected organ systems, including the nervous system (in 2 patients, one of which is the index patient), lungs (5 patients), eyes (3 patients), liver (1 patient), and skin (2 patients). All but one had pathology-confirmed sarcoidosis. Ingenuity Variant Analysis™ filtering resulted in 358 candidate variants within 132 different genes in the six affected family members with a potential association with development of sarcoidosis. Supplementary figure 1 shows our step-by-step workflow of selection of candidate genes. We developed primers for AmpliSeq™ to sequence and evaluated 294 of the candidate variations leading to 36 variants within 34 genes passing the initial filtering (Table 2, Supplementary figure 1). Pathway analysis showed no associations of the 34 genes and overrepresentation of a specific pathway. This means that no general pathway within the reported relevant variations is detected to explain the disease cause. The genes with the highest minor allele frequency (MAF) in affected versus unaffected patients were ZFAT (MAF affected/unaffected, 4.57), DNAH9 (MAF affected/unaffected, 3.43), NCF1 (MAF affected/unaffected, 3.43), and MCM2 (MAF affected/unaffected, 3.24). The genes with the highest CADD scores were OTOG (28.5), MYF6 (27.7), and NUN6 (26.6). In total, 5 genes had a CADD score below 1, consisting of SETSIP, EHMT1, MUC5B, CSH1/CSH2, and BIRC5. Furthermore, evaluation with the ClinVar database did not result in additional information.

**Table 2:** Variants of interest

#	Chr	Location^a^	Rs number	Gene^b^	Ref	Var	MAF A^c^	MAF U^d^	MAF A/U	ExAC database allele frequency	ClinVar database	CADD score
1	1	1049746	rs199876002	AGRN	G	C	0.286	0.219	1.306	0.00358	A	14.78
2	1	92075441	rs139959666	SETSIP	T	C	0.429	0.188	2.286	-	NR	0.015
3	1	192810219	rs140811638	RGS2	C	T	0.286	0.141	2.032	0.0000498	NR	24.4
4	1	207645313	rs115047031	CR1L	T	G	0.286	0.103	2.776	0.00352	NR	15.96
5	3	127619115	rs149699620	MCM2	C	T	0.286	0.088	3.238	0.000746	NR	7.279
6	5	142314552	rs148983803	SPRY4	C	T	0.357	0.250	1.429	0.00253	A	25.7
7	6	7580645	rs113902911	DSP	G	T	0.429	0.172	2.494	0.00175	A	17.66
8	6	89950854	rs34335140	BACH2	C	T	0.214	0.203	1.055	0.00197	A	17.98
9	6	160079596	rs8191904	IGF2R	G	T	0.214	0.125	1.714	0.00324	A	9.852
10	7	74782983	rs782555266	NCF1	A	G	0.214	0.063	3.429	-	NR	7.537
11	7	129205670	rs111694017	SMO	G	A/A	0.429	0.206	2.082	0.00741	A	18.27
12	8	60795119	rs141947938	CHD7	G	A	0.286	0.109	2.612	0.00128	A	24.4
13	8	60853203	rs61753399	CHD7	G	A	0.286	0.118	2.429	0.00128	A	12.52
14	8	134590348	rs138033806	ZFAT	C	T	0.214	0.047	4.571	0.00320	NR	20.6
15	9	5055741	rs149683525	JAK2	A	G	0.357	0.203	1.758	0.000369	U	16.13
16	9	137244204	rs76517216	FAM166A	C	T	0.357	0.221	1.619	0.00235	NR	23.8
17	9	137813149	rs138283222	EHMT1	G	A	0.286	0.188	1.524	0.000250	A	0.042
18	10	18551939	rs139589125	NSUN6	G	C	0.214	0.094	2.286	0.0000330	NR	26.6
19	10	19146262	rs111530988	MALRD1	T	G	0.214	0.103	2.082	-	NR	2.860
20	10	77977594	rs147168447	POLR3A	T	C	0.286	0.141	2.032	0.000140	U	22.0
21	11	1241237	rs56079125	MUC5B	G	A	0.500	0.221	2.267	0.00145	NR	0.008
22	11	17610849	rs376684690	OTOG	C	G	0.286	0.172	1.662	0.000833	A	13.27
23	11	17631882	rs142799217	OTOG	G	A	0.286	0.206	1.388	0.000309	A	28.3
24	11	47259902	rs41481445	NR1H3	G	T	0.286	0.188	1.524	0.00235	A	1.067
25	11	128489374	rs79963544	ETS1	C	T	0.286	0.221	1.295	0.00231	A	21.4
26	12	80708548	rs141278987	MYF6	A	C	0.286	0.118	2.429	0.000685	A	27.7
27	13	25097753	rs757885062	PABPC3	C	T	0.214	0.074	2.914	0.0000247	NR	10.23
28	13	28311998	rs145693340	FLT1	G	T	0.214	0.076	2.829	0.000173	NR	24.1
29	13	32239736	rs193120945	FRY	A	G	0.214	0.088	2.429	0.00228	A	23.1
30	17	11894431	rs78870819	DNAH9	C	T	0.214	0.063	3.429	0.000981	NR	23.4
31	17	39526122	rs56362165	CDK12	T	A	0.214	0.162	1.325	0.00220	U	13.64
32	17	63896505	rs1130686	CSH1/CSH2	G	C	0.286	0.266	1.076	0.000512	NR	0.026
33	17	75625192	Unknown	MYO15B	G	C	0.214	0.088	2.429	-	U	26.5
34	17	78223530	rs17880183	BIRC5	C	T	0.286	0.203	1.407	0.00208	NR	0.354
35	22	15528887	rs141845380	OR11H12	C	T	0.214	0.156	1.371	0.000371	NR	4.512
36	X	37795981	Unknown	CYBB	A	T	0.222	0.204	1.089	-	U	22.6

^a^Locations reported are according to human assembly GRCh38/hg38 build

^b^Two additional variants were identified (3:20558901, ELK4 and 3:113376110, USF3). Both consisted of deletions; however, the used methods were not appropriate to evaluate deletions and duplications

^c^Affected

^d^Unaffected

*NR*, not reported; *A*, benign/likely benign; *U*, uncertain significance

From the 34 discovered genes, 14 have previously been described to have a role in the immune system. A total of 6 genes are involved in the adaptive immune system, including T and B cell activation or proliferation (RGS2, AGRN, ETS1, IGF2R, SMO, and ZFAT) and 3 have a role in the innate immune system, including dendrocyte and/or macrophage activation or migration (POLR3A, NR1H3, and FLT1) [7, 8]. Additionally, BACH2 codes for a transcription factor involved in both the macrophage-mediated innate immune response and the adaptive immune response [9]. Furthermore, four genes have various roles directly linked to the immune system. CR1L is involved in the complement system (CR1L) [10]. NCF1 and the x-chromosomal CYBB code for a subunit of neutrophil nicotinamide adenine dinucleotide phosphate (NADPH) oxidase, which is a membrane-bound enzyme complex that generates superoxide [11]. JAK2 codes for a protein tyrosine kinase, which is required for responses to interferon gamma (IFNγ), granulocyte-macrophage colony-stimulating factor (GM-CSF), and several interleukins (IL-3, IL-5, IL-6, and IL-12, among others) [12].

Of the remaining 20 genes, 18 genes have functions that have not been associated with the immune system and two genes have an unknown function. Six genes are involved in the cardiovascular system (SETSIP), respiratory tract (DNAH9), digestive tract (MALRD1), ear and nose (OTOG and OR11H12), and musculoskeletal system (MYF6) [13–17]. Additionally, DSP codes for desmoplakin and is involved in cell structure in cardiac tissue and epidermal cells and MUC5B codes for the components of mucus and is mainly expressed in the lungs [18, 19]. Ten genes have various roles in the cell cycle, mainly cell division and proliferation (FRY, BIRC5, CHD7, and EHMT1), but also DNA replication, cell cycle regulatory proteins and genomic stability, and repair (CDK12, SPRY4, and MCM2, respectively) [20, 21]. Also, two genes have roles in RNA modification and translation initiation (NSUN6 and PABPC3) and CSH1 codes for a protein that is only secreted during pregnancy and stimulates fetal growth [22, 23]. Of the remaining two genes, the exact function is unknown (MYO15B and FAM166A).

## Discussion

We identified 36 variants in 34 genes with possible roles associated with sarcoidosis in a large family with a high prevalence of the disease. We identified genetic variations in JAK2, BACH2, and NCF1 that have been associated with autoimmune diseases such as inflammatory bowel disease and chronic granulomatous disease [24, 25].

Identification of the JAK/STAT pathway is of special interest because of potential clinical use of JAK-specific inhibitors in sarcoidosis [26]. JAK2 codes for a protein tyrosine kinase, which is required for responses to interferon gamma (IFNγ), granulocyte-macrophage colony-stimulating factor (GM-CSF), and several interleukins (IL-3, IL-5, IL-6, and IL-12, among others) [12]. Both IFNγ and GM-CSF have been associated with sarcoidosis and disease activity [27, 28]. Upon activation, JAK2 phosphorylates signal transducer and activator of transcription (STAT) and initiates the JAK/STAT signaling pathway. The JAK/STAT pathway has been implicated in both the IL-12 and IL-23 signaling pathways, which leads to Th1 and Th17 CD4 cell maturation [12]. These Th cells are important mediators of immune responses and are thought to organize the granulomatous structure, which is in turn highlighted by CD4 T cell lymphopenia in sarcoidosis patients [29]. In a study evaluating microRNA expression and protein-coding gene expression in sarcoidosis patients, they found the JAK/STAT signaling pathway to be the most significantly involved pathway [30]. A genetic association study involving 1996 German sarcoidosis patients described an overlap between risk loci in inflammatory bowel disease and sarcoidosis, especially in the IL-23 signaling pathway [31]. Authors described two variants in the JAK2 gene associated with chronic sarcoidosis [31].

JAK is a novel therapeutic target in inflammatory bowel disease. The two major forms of inflammatory bowel disease, Crohn’s disease and ulcerative colitis, are also chronic immune-mediated conditions characterized by an increased production of pro-inflammatory cytokines leading to granuloma formation [26]. In line with our findings in sarcoidosis patients, genetic association studies have linked inflammatory bowel disease to the JAK/STAT pathway [32]. In two randomized clinical trials, involving patients with moderately to severely active ulcerative colitis, tofacitinib was more effective as induction and maintenance therapy than a placebo [26]. Tofacitinib, a pan-JAK inhibitor, has recently been approved for the treatment of moderate-to-severe ulcerative colitis [33]. Furthermore, positive effects of tofacitinib have been described in patients with sarcoidosis [34]. We previously described that one-third of patients with neurosarcoidosis do not respond to treatment [2]. Future research might evaluate JAK inhibitors in patients with treatment-resistant sarcoidosis.

BACH2 codes for a transcription factor involved in both the macrophage-mediated innate immune response and the adaptive immune response. It is expressed in B and T cells, alveolar macrophages, and neural cells [9]. BACH2 is a broad regulator of immune activation and is required for the formation of regulatory T cells. It has an essential role in maintaining immune homeostasis [9,3 5]. Furthermore, it limits the Th1 and Th17 differentiation to effector lineages CD4+ T cells [35]. In mice knocked out for BACH2, investigators found an increase in the number of CD4 T cells in the lungs and peripheral lymphoid organs and increased proportions of effector cells in the lungs. As described above, these CD4 T cells play an important role in granuloma organization. Most notably, variations in the BACH2 gene are associated with Crohn’s disease [24]. Only one study found a possible role for BACH2 in sarcoidosis patients. They found a negative correlation between gene expression of BACH2 and disease severity [30].

Variants in NCF1 were one of the most distinctive variations, which were more frequently found in affected family members. NCF1 codes for a 47-kDa cytosolic subunit of neutrophil NADPH oxidase. Mutations in this gene lead to a lower level of reactive oxygen species leading to decreased intracellular killing in phagocytic cells [11]. NCF1 is associated with chronic granulomatous disease. Patients with this disease have increased susceptibility to recurrent infections and granuloma formation. We have identified an additional candidate gene, the x-chromosomal CYBB, with a common pathway, also associated with this disease. NCF1 mutations have also been associated with an increased susceptibility for systemic lupus erythematosus (SLE), Sjögren’s syndrome, and rheumatoid arthritis [25]. Sarcoidosis in patients with chronic granulomatous disease has been described, but is rare which might be due to underreporting given the difficulty to discriminate between the two diseases [36]. Animal studies have shown that NCF1-mutated mice show an increase of T cell–dependent autoimmunity [37]. Furthermore, NAHDP deficiency may play a role in decreased antigen degradation and increased cross-presentation of antigens via MHC class I or II between dendritic cells, possibly enhancing auto-immunity [38]. In mice with a loss of NAHDP function, an increase of non-caseating granuloma formation was observed in response to *Propionibacterium acnes* [39]. Previous studies have demonstrated the occurrence of microbes in patients with sarcoidosis leading to the hypothesis that decreased bacterial clearance may add to granuloma formation [40].

Sarcoidosis is thought to be a complex disease in which many genetic variations with small effect are involved in addition to unknown environmental factors [1]. Multiple variants have been found in genome-wide association studies and replication of these results proved to be difficult, as illustrated by HLA-DR9 which has been associated with disease risk in Japanese patients, but with disease protection in Scandinavians [41]. It has been suggested that population stratification and failure to correct for population-specific or ethnicity-specific traits remain a limitation and this might be improved when cohorts are stratified on the basis of genetically determined ancestry [42]. Family-based studies have the capacity to limit the confounding ancestry-specific genetic predisposition. For example, early family linkage studies have linked a familial risk to the MHC region, which have further been pinpointed to haplotype variation in the region of the BTNL2 gene with the strength of the association depending on ethnicity [43]. The advantage of our approach, which is performed within a family with affected individuals with a shared ancestry, is that it can focus on rare genetic triggers with strong effects, keeping in mind the polygenetic nature of the disease. Recently, three family studies were published evaluating genetic predisposition using a comparable approach [44–46]. A whole exome sequencing study in 14 affected individuals and 8 unaffected family members in 5 different families used prediction software and a minor allele frequency lower than 0.05 to further select candidate genes and found 227 variants in 192 genes. Analysis of the involved pathways identified the TOR pathway as a possible association. No information on the ethnic background or admixture of the selected families was published. Another whole exome sequencing study in 22 affected German patients from 6 families and 14 from 5 families did not use healthy family members as a control group. They looked at shared genetic predisposition between the families and variants were filtered if they occurred in less than 50% of each pedigree. They found 40 candidate variants involved in Wnt signaling, chemokine- and cytokine-mediated inflammatory responses and cadherin signaling pathways. Interestingly, no commonly shared genomic region or gene was identified among the analyzed six sarcoidosis pedigrees, highlighting the complex and heterogeneous genotype-phenotype relation of sarcoidosis. A third study investigated traits associated with disease onset before the age of 15 in patients and used their healthy parents as controls. They selected variants that only occurred in affected patients or were transmitted as recessive traits from each parent. Ethnic background was not provided. They found 37 candidate genes, but none was shared by all patients. Altogether, many genetic variations are suggested to be of influence in sarcoidosis, but few are replicated in a population with shared ethnic ancestry. Further exploration in multiple families with a shared ancestry with high prevalence of sarcoidosis is an important next step in future studies to identify candidate variants and genes.

Our study has several limitations. First, DNA was collected from a large number of family members but not all. Second, clinical data could not be collected in all patients especially in the first two generations as the diagnosis was made more than 20 years ago, subjects died, or were living abroad. In the individuals of whom data could not be collected, clinical information was verified by family members, which may be unreliable. However, the diagnosis was confirmed by biopsy in 67% of the individuals that limits the diagnostic uncertainty. Third, patients of the fourth and fifth generations may still develop sarcoidosis due to their relatively young age. Fourth, we cannot exclude that there is a non-genetic shared exposure to unknown antigens in addition to a shared genetic risk leading to a bias in our results. However, this cannot be overcome in a family study. The size of the family including different generations living in different parts of The Netherlands and Suriname is expected to limit shared exposure to unknown antigens. Fifth, the presence of variations within other populations is an important filter in our analysis to selected candidate variations associated with sarcoidosis. The ideal control population for this family of Suriname origin would have been one with African-Caribbean descent. With the exception of the Barbados 1000 genome project population, African-Caribbean’s populations are underrepresented in population genetics, which could have affected the selection of associated variations [47]. Currently, no other African-Caribbean sarcoidosis cohorts or families have been described making validation of our results difficult and we could not investigate the role of these variants in the pathogenesis of sarcoidosis. In line with autoimmune disease GWAS, we do not find a single causal gene but multiple candidate genes within the family for sarcoidosis indicating a multifactorial genetic origin for this disease. This indicates the complexity of detecting the involved genetic variability in sarcoidosis risk and possible correlation with the highly variable presentation and progression of the disease [4]. Lastly, IVA does not target certain structural variants and deletions and duplications might be missed in our analysis.

In this family-based study, we have identified 36 variants in 34 candidate genes with a possible role in the etiology of sarcoidosis. Identified variations within three of these genes, namely JAK2, BACH2, and NCF1, have previously been associated with multiple autoimmune diseases. Of special interest is the role of JAK/STAT pathway in the pathophysiology of sarcoidosis. JAK inhibitors have been approved for colitis ulcerosa, a disease with many similar aspects in the pathophysiology, so this may be a novel treatment option for treatment-resistant sarcoidosis.

## Supplementary information

Supplementary figure 1. Flow chart of variant selection (DOCX 168 kb)

## Data Availability

Anonymized data not published in the article is available on request by any qualified investigator.
