# Supplementary material for: Whole genome sequencing identifies variants associated with sarcoidosis in a family with a high prevalence of sarcoidosis
Source: Clin Rheumatol. 2021 Apr 27;40(9):3735–43. doi: 10.1007/s10067-021-05684-w (PMC8357727; doi:10.1007/s10067-021-05684-w)
Supplement: Supplementary file 1 — Flow chart of variant selection (DOCX 168 kb) [file 10067_2021_5684_MOESM1_ESM.docx]

**SUPPLEMENTARY MATERIAL**

**SUPPLEMENTARY FIGURES**

**Supplementary figure 1. Flow chart of variant selection**

**
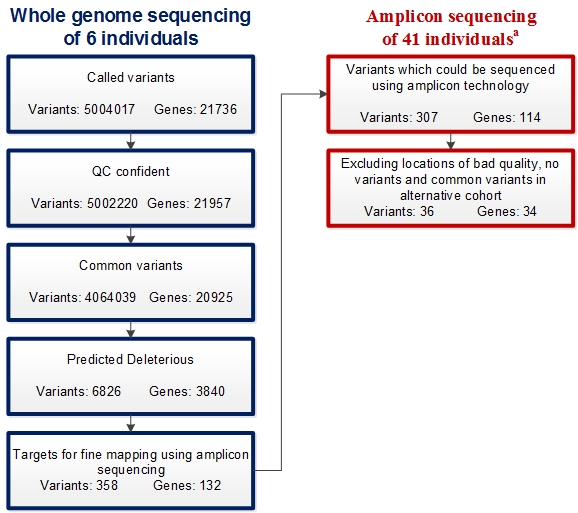
**

^a^ The 6 whole genome sequenced individuals are included in the 41individuals who were amplicon sequenced.
